# Comparison of community and clinic-based blood pressure measurements: A cross-sectional study from Haiti

**DOI:** 10.1371/journal.pgph.0001064

**Published:** 2022-09-30

**Authors:** Caleigh E. Smith, Miranda Metz, Jean Lookens Pierre, Vanessa Rouzier, Lily D. Yan, Rodney Sufra, Eliezer Dade, Fabyola Preval, Wilson Ariste, Vanessa Rivera, Olga Tymejczyk, Rob Peck, Serena Koenig, Marie Marcelle Deschamps, William Pape, Margaret L. McNairy

**Affiliations:** 1 Department of Medicine, Center for Global Health, Weill Cornell Medicine, New York, New York, United States of America; 2 Haitian Group for the Study of Kaposi’s Sarcoma and Opportunistic Infections (GHESKIO), Port-au-Prince, Haiti; 3 Division of General Internal Medicine, Department of Medicine, Weill Cornell Medicine, New York, New York, United States of America; 4 City University of New York Institute for Implementation Science in Population Health, New York, NY, United States of America; 5 Department of Medicine, Brigham and Women’s Hospital, Boston, Massachusetts, United States of America

## Abstract

Hypertension (HTN) is the leading modifiable cardiovascular disease (CVD) risk factor in low and middle-income countries, and accurate and accessible blood pressure (BP) measurement is essential for identifying persons at risk. Given the convenience and increased use of community BP screening programs in low-income settings, we compared community and clinic BP measurements for participants in the Haiti CVD Cohort Study to determine the concordance of these two measurements. Participants were recruited using multistage random sampling from March 2019 to August 2021. HTN was defined as systolic BP (SBP) ≥ 140mmHg, diastolic BP (DBP) ≥ 90mmHg or taking antihypertensives according to WHO guidelines. Factors associated with concordance versus discordance of community and clinic BP measurements were assessed with multivariable Poisson regressions. Among 2,123 participants, median age was 41 years and 62% were female. Pearson correlation coefficients for clinic versus community SBP and DBP were 0.78 and 0.77, respectively. Using community BP measurements, 36% of participants screened positive for HTN compared with 30% using clinic BPs. The majority of participants had concordant measurements of normotension (59%) or HTN (26%) across both settings, with 4% having isolated elevated clinic BP (≥140/90 in clinic with normal community BP) and 10% with isolated elevated community BP (≥140/90 in community with normal clinic BP). These results underscore community BP measurements as a feasible and accurate way to increase HTN screening and estimate HTN prevalence for vulnerable populations with barriers to clinic access.

## Introduction

Hypertension (HTN) is the leading risk factor for cardiovascular disease (CVD) globally, with an estimated 1.28 billion adults affected, two-thirds of whom live in low-and middle-income countries (LMICs) [1]. The World Health Organization (WHO) has prioritized a goal of reducing HTN prevalence 25% by 2025 to reduce CVD-related morbidity and mortality [2]. Access to accurate blood pressure (BP) measurement is critical to identify individuals with elevated BP who need follow-up for diagnosis, counseling, and treatment, as control of elevated BP in LMICs is particularly poor [3]. A multinational cross-sectional study found that awareness, treatment and control of BP was lowest in LMICs, with approximately one third of persons with HTN receiving treatment and only 10% being adequately controlled with medication [3]. Community-based BP screening is an increasingly common strategy to expand screening among such vulnerable populations with limited access to healthcare [4–6].

HTN appears to be the most common CVD risk factor in Haiti [7, 8], with the most recent 2016–2017 Demographic Health Survey (DHS) reporting a HTN screening prevalence of 49% in women and 40% in men, ages 35–64 years [7]. In addition, the onset of HTN in Haiti appears to occur 10–20 years earlier than among Black Americans in the US [8–10]. Access to healthcare services is a significant barrier to HTN screening, diagnosis, and treatment in Haiti, with poverty and political insecurity preventing many patients from seeking care [9, 11–13]. Therefore, the expansion of community-based HTN screening and diagnosis in Haiti is crucial to working towards the WHO’s goal of reducing HTN prevalence and related CVD mortality.

There are limited data comparing community and clinic BP measurements in LMICs despite extensive research from developed countries suggesting that BP measurements taken outside the clinic differ from clinic-based measures in many populations [14–18]. In this analysis, we compare community- and clinic-based BP measurements among a cohort of participants in the Haiti CVD Cohort Study who had both measurements taken during study enrollment procedures [19]. The goal of this analysis is to compare community and clinic BP measurements and to determine the concordance of BP measurement classification across these setting. These findings will have actionable implications for estimating HTN prevalence as well as for identifying patients with HTN who need treatment in Haiti, with applications in other settings with limited healthcare access.

## Methods

### Ethics statement

The study protocol and ethical consent forms were approved by the institutional review board at Weill Cornell Medicine and the Ethics Board at GHESKIO (record number 1803019037). Written consent was obtained for all participants.

### Study site

This study was conducted at the Groupe Haïtien d’etude du Sarcome de Kaposi et des Infections opportunists (GHESKIO) in Port-au-Prince, Haiti. GHESKIO is a large medical facility that was founded in 1982 to provide clinical care for infectious diseases, and more recently, non-communicable diseases. Since 2015, GHESKIO has expanded their community HTN screening efforts. GHESKIO community health workers (CHWs) are trained to measure BP using electronic sphygmomanometers and conduct BP counseling in communities across downtown Port-au-Prince.

### Study design and population

We performed a cross-sectional analysis within the Haiti CVD Cohort Study, a population-based longitudinal cohort study of adults living in metropolitan Port-au-Prince (clinicaltrials.gov #NCT03892265). The Haiti CVD Cohort Study was designed to estimate prevalence and incidence of CVD and its risk factors via methods described in detail previously [19]. The study population includes individuals recruited from households using multistage random sampling. Inclusion criteria were age ≥18 years, primary residence in Port-au-Prince, and ability to speak and understand French or Creole. Participants were enrolled from March 19, 2019 to August 23, 2021.

### Study procedures

#### Blood pressure measures.

All study BP measurements were performed according to WHO and American Heart Association (AHA) guidelines [20, 21]. Individuals were seated for five minutes prior to BP measurement with the left arm supported at heart level, both feet on the ground, and use of an appropriate cuff size corresponding to arm circumference. Three BP measurements were taken, each separated by 30 seconds, and the mean of the last two measurements was taken as the BP measurement for this analysis [19, 22].

Community BP measurement was performed prior to clinic BP for all participants. During recruitment in the community, trained CHWs identified randomly selected households and generated a roster of all adults living in each household. Following verbal consent, CHWs measured community BP for all adults present in the home at the time of the survey using an Omron BP742N 5-series electronic cuff [23]. Participants were then randomly selected from the household roster for participation in the study and invited to GHESKIO for written informed consent and enrollment procedures. During enrollment at GHESKIO, clinic BP was measured by a research nurse or nursing aid with an Omron HEM-907 series sphygmomanometer.

#### Other measures and outcomes.

Sociodemographic, clinical, and health behavior measures were collected at time of study enrollment at GHESKIO. Sociodemographic data include age, sex, education, and income. Clinical and health behavior data include height, weight, and smoking status. Education was categorized as having completed no education, primary, or secondary/higher. Income was categorized as ≤ 1 USD per day or > 1 USD per day, with 1 USD corresponding to 100 Haitian gourdes at the time of analysis. Participants were categorized into body mass index (BMI) groups based on WHO guidelines, where < 18.5 kg/m^2^ was defined as underweight, 18.5 to 24.9 kg/m^2^ as normal weight, 25.0 to 29.9 kg/m^2^ as overweight, and ≥30.0 kg/m^2^ as obese [20]. Participants were categorized as never smokers or current/former smokers.

In this analysis, definitions of elevated BP categories follow WHO guidelines, which are widely used in LMICs and differ from the most recent AHA guidelines [20, 21]. HTN was defined as SBP ≥ 140 mmHg and/or DBP ≥ 90 mmHg, or a self-report of taking anti-hypertensive medications in the last two weeks [20]. Participants who self-reported taking antihypertensive medications were categorized as having hypertension due to a previous diagnosis, regardless of their blood pressure. Normal BP was defined as SBP < 120 mmHg andDBP < 80 mmHg. Pre-hypertension was defined as SBP 120–139 mmHg or DBP 80–89 mmHg. Stage 1 HTN was defined as SBP 140–159 mmHg or DBP 90–99 mmHg. Stage 2 HTN was defined as SBP ≥ 160 mmHg or DBP ≥ 100 mmHg. Age adjusted HTN screening prevalence was calculated based on both community and clinic BP measurements using the World WHO 2000–2025 Standard Population [24].

Elevated community BP was defined as a community BP measurement SBP ≥ 140 or DBP ≥ 90 and clinic BP measurement SBP <140 and DBP < 90. Elevated clinic BP was defined as a clinic measurement with SBP ≥ 140 and DBP ≥ 90 and community BP measurement with SBP < 140 and DBP < 90. Concordant normotension was defined as both clinic and community measurements with SBP < 140 and DBP < 90. Concordant HTN was defined as clinic SBP ≥ 140 or DBP ≥ 90 mmHg and community SBP ≥ 140 or DBP ≥ 90 mmHg, or a self-report of taking anti-hypertensive medications.

Sensitivity analyses were conducted to evaluate the prevalence of elevated clinic and community BP using cut-offs of SBP ≥ 130 mmHg or DBP ≥ 80 mmHg at home and in the clinic in accordance with the most recent AHA guidelines [21].

### Statistical analysis

This analysis was restricted to participants with information on sex, age, and BP measurements from both the home and clinic. Descriptive statistics were generated for these study participants according to the definitions described above. We compared median BP and proportions of pre-hypertension and HTN for all participants based on community and clinic BP measures. Associations between community and clinic SBP and DBP measurements were plotted using a linear regression with 95% confidence intervals (CI), and correlations were estimated using Pearson coefficients. Differences between community and clinic BP measurements were calculated by subtracting community BP from clinic BP (mmHg) for each participant. Two-sided t-tests were used to compare the difference in mean SBP between males and females. The relationship between days between BP measurements in the community and clinic settings with the difference between SBP and DBP was assessed using Pearson coefficients.

We estimated the proportion of participants with concordant classification of normotension or HTN in both the clinic and the community as well as the proportion of discordance with elevated BP in either the community or clinic alone. Multivariable Poisson regressions were used to assess for factors associated with isolated elevated BP in the clinic, isolated elevated BP in the community, and concordant HTN compared to participants with concordant normotension. For each model, the covariates included age category, sex, BMI category (normal/underweight, overweight, obese), education level (none, primary, secondary, or higher), income (> 1 USD or ≤ 1 USD), and smoking status (never or current/former). Robust standard errors were used for all regression models.

All analyses were performed using R statistical software, version 1.2.5019.

### Inclusivity in global research

Additional information regarding the ethical, cultural, and scientific considerations specific to inclusivity in global research is included in the (S1 Checklist).

## Results

### Baseline characteristics and blood pressure

Of 3,005 participants enrolled, 2,123 (71%) had both community and clinic BP measurements and were included in this analysis. The median number of days between community and clinic BP measurements was 4 (interquartile range [IQR]: 2–11). Median age was 41 years (IQR: 28–55), and 1,320 (62%) participants were female (Table 1). A total of 792 (37%) reported only primary schooling or no education, and 1511 (71%) earned less than 1 USD/day. Only 182 (9%) were smokers, 580 (28%) were overweight, and 392 (19%) were obese.

A total of 770 (36% crude, 35% age standardized) participants screened positive for HTN using a community BP measurement as compared to 644 (30% crude, 29% age standardized) using a clinic BP measurement (Table 2). Using sensitivity analysis cutoffs of SBP ≥ 130 mmHg or DBP ≥ 80 mmHg, 1174 (55%) participants screened positive for HTN based on the community measurement, and 897 (42%) participants screened positive for HTN based on the clinic measurement. There were 553 participants (26%) with pre-HTN based on community BP measurement compared to 455 participants (21%) in clinic. Three hundred and eighty-five participants (18%) had at least one BP measurement (community or clinic) above 160/100 mmHg (Stage 2 HTN). There were 291 participants with Stage 2 HTN in the community, of whom 152 (52%) remained Stage 2 based on the clinic measurement, and 251 (86%) were at least Stage 1 in the clinic.

**Fig 1.**
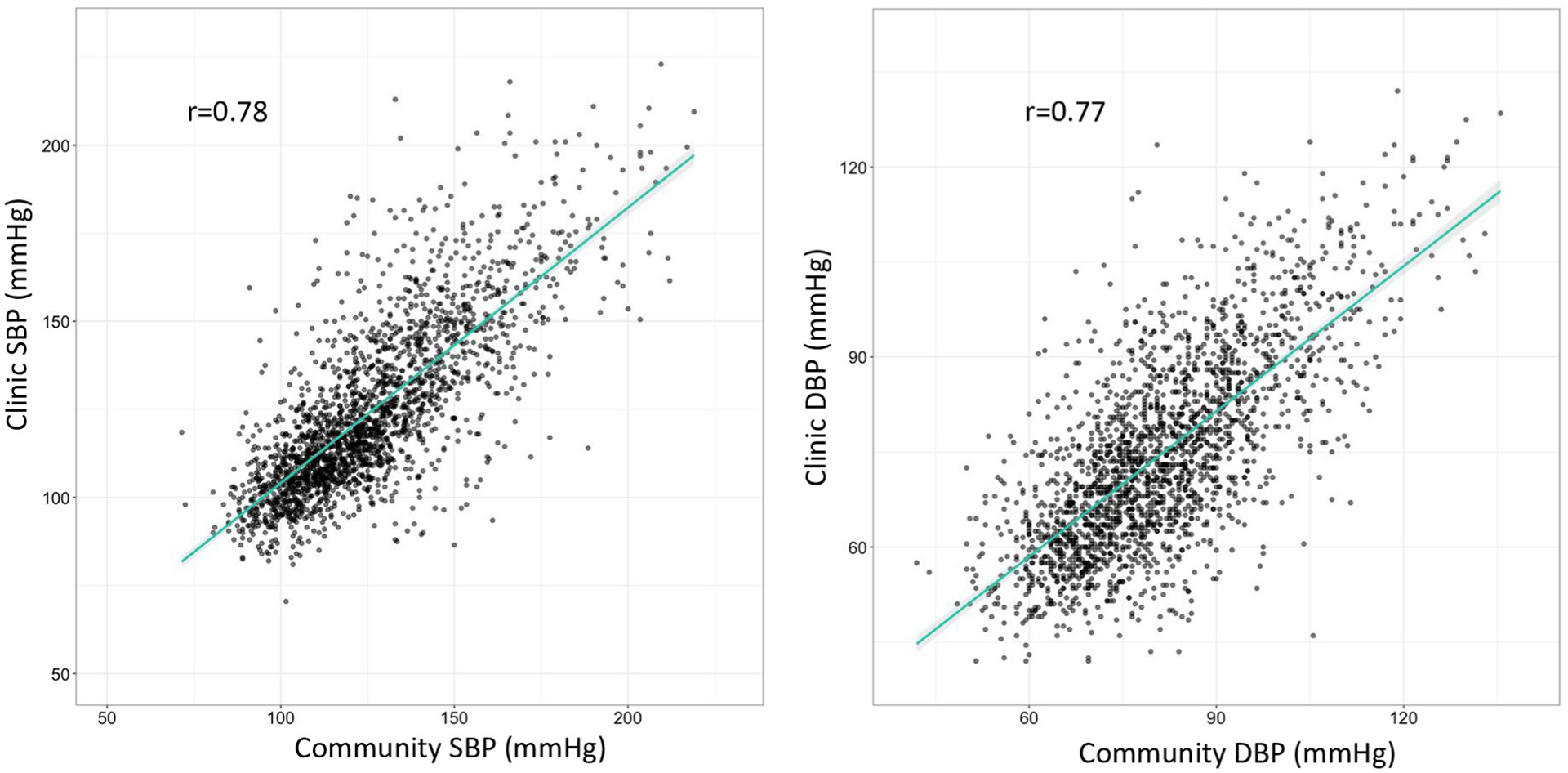
Correlation of systolic and diastolic blood pressure in mmHg in the clinic versus community measurement (N = 2123). The line represents the linear regression between clinic and community BP.

**Fig 2.**
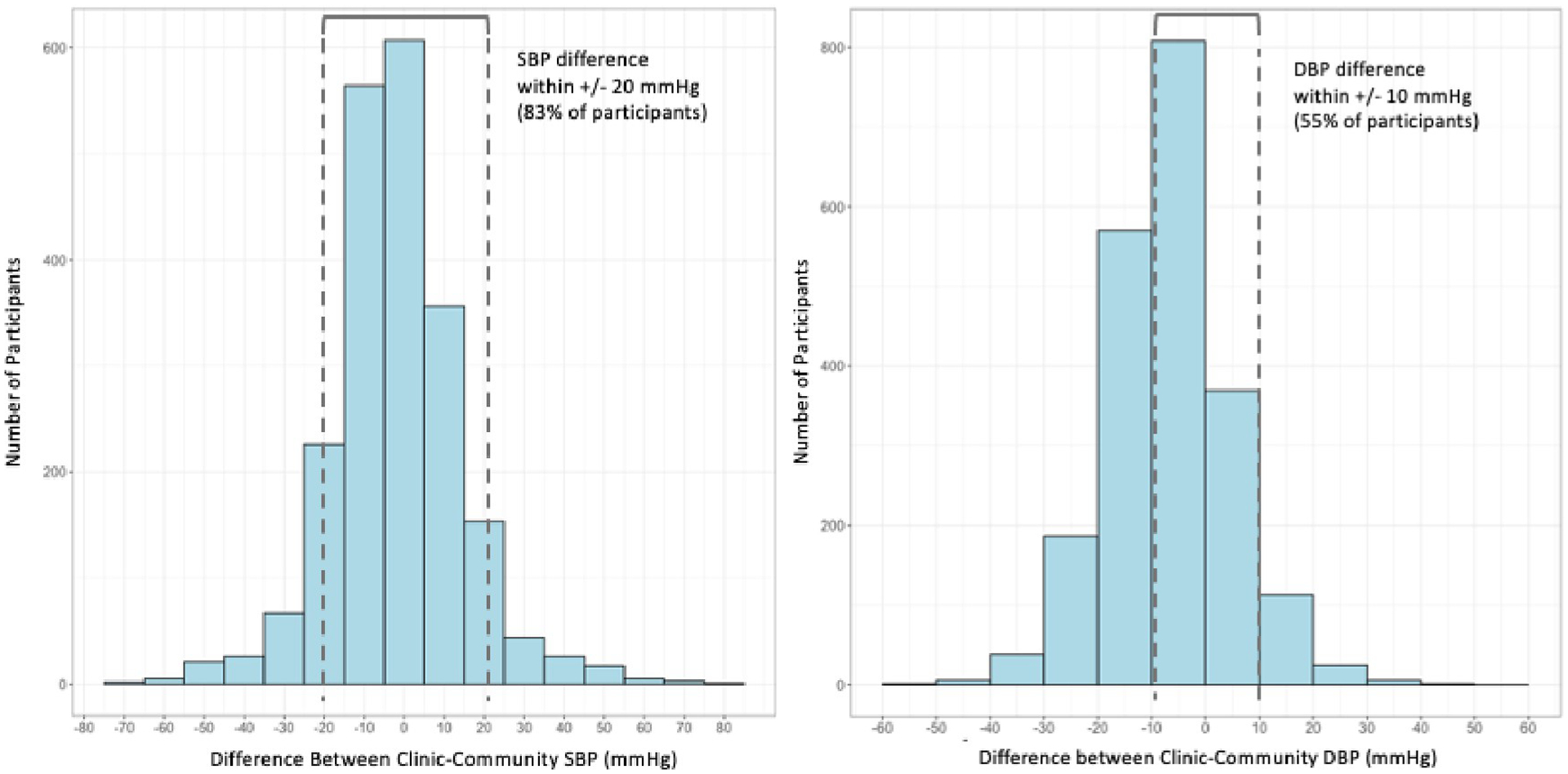
Difference in systolic and diastolic blood pressure between a participant’s clinic and community measurements (N = 2123).

The median community SBP was 122 mmHg (IQR: 109–140), and the median clinic SBP was 118 mmHg (IQR: 107–138). The median community DBP was 80 mmHg (IQR: 71–90) as compared to median clinic DBP of 73 mmHg (IQR: 63–85). Median community and clinic BP measurements did not differ significantly by sex. The Pearson correlation coefficients for clinic versus community SBP and DBP were 0.78 and 0.77, respectively (Fig 1). Fig 2 illustrates the difference between a participant’s clinic and community SBP and DBP measurements (clinic minus community). There were 1,757 participants (83%) with a difference between community and clinic SBP within +/−20 mmHg, and 1,170 participants (55%) had a DBP difference within +/−10 mmHg. The median difference in SBP (clinic-community) was −2.0 mmHg (IQR: −10.5 to 7.0), and the median difference in DBP (clinic-community) was −6.5 mmHg (IQR: −13.5 to 0). Mean clinic SBP in males was 127.2 mmHg as compared to 123.0 mmHg for females (p-value < 0.001). Mean community SBP in males was 129.6 mmHg for males versus 124.1 mmHg for females (p-value < 0.001). Males had a greater mean difference in clinic-community BP measurements than females: of −2.39mmHg and −1.16mmHg for females (p = 0.08). There was no relationship between the time between measurements and the SBP difference (p = 0.45) or DBP difference (p = 0.87) between clinic and community measures.

#### Concordance of community and clinic blood pressure measurement status.

**Fig 3.**
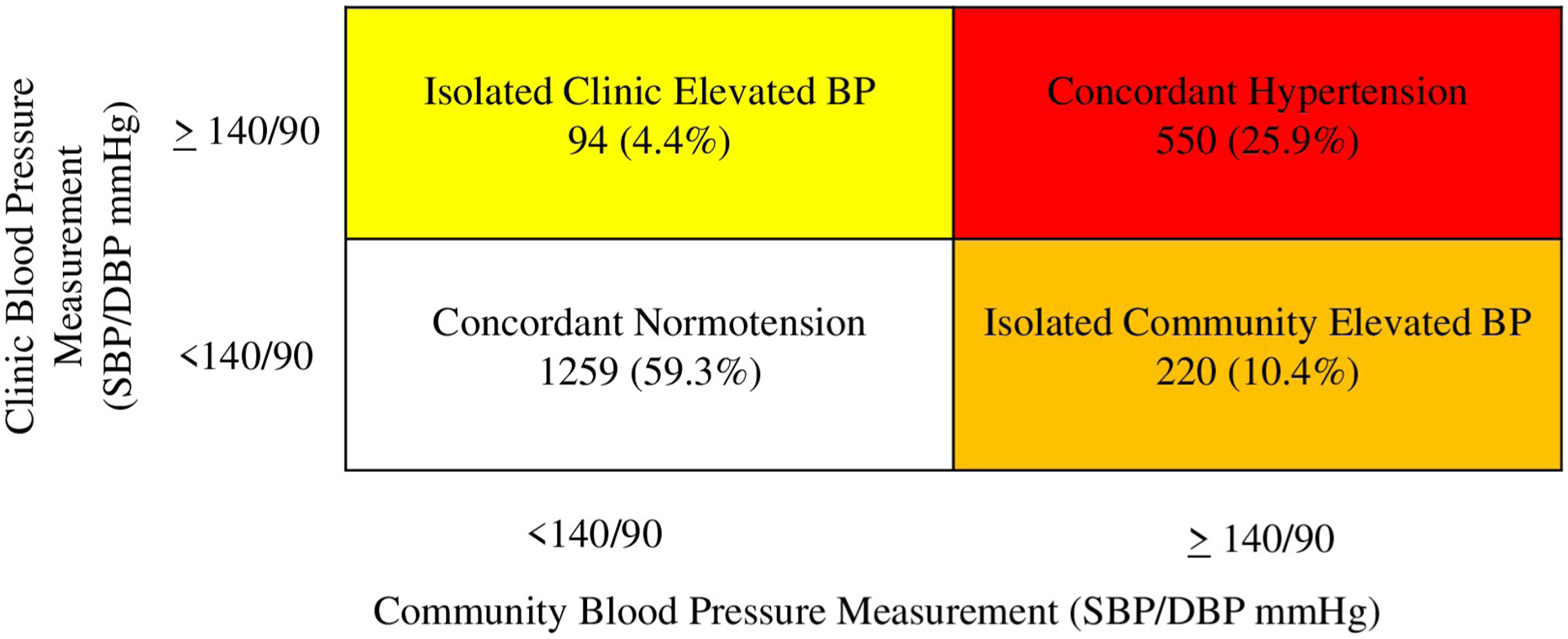
Classification of blood pressure status categorization according to clinic and community measurements (N = 2123). Concordant Normotension: BP <140/90 at home and at the clinic measurement. Concordant Hypertension: BP ≥ 140/90 at home measurement and at the clinic measurement OR the participant reports taking antihypertensive medication (regardless of the BP in either setting). Isolated Clinic Elevated BP: BP <140/90 at community measurement and BP ≥ 140/90 at the clinic measurement. Isolated Community Elevated BP: BP ≥ 140/90 at community measurement and BP <140/90 at the clinic measurement.

Using both community and clinic BP measurements, a total of 85% of participants had concordant BP status classification across measurements, with 1,259 adults (59%) having concordant normotension and 550 (26%) having concordant HTN (Fig 3). Ninety-four participants (4%) had isolated elevated BP at the clinic, and 220 participants (10%) had isolated elevated BP at the community. In the sensitivity analysis using lower AHA thresholds [21], 95 participants (4%) had isolated elevated clinic BP, and 372 participants (18%) had isolated elevated community BP.

Of the 550 adults with concordant HTN, the median age was 57 (IQR: 48–63). The median community BP was SBP 150 mmHg/DBP 94 mmHg, and the median clinic BP was SBP 151 mmHg/DBP 91 mmHg. Median community SBP was 0.5 mmHg higher and median DBP 4.0 mmHg higher than corresponding clinic measures. Among 550 participants with concordant HTN, 273 (50%) reported use of antihypertensive medication. The median community BP for those taking antihypertensives was SBP 144 mmHg/DBP 90 mmHg, and the median clinic BP was SBP 148 mmHg/DBP 88 mmHg. Among participants who reported taking antihypertensives, 64% had a BP ≥ 140/90 mmHg at community compared with 69% at the clinic.

Of the 94 participants with isolated elevated BP at the clinic, the median age was 53 (IQR: 43–64), and 64 (68%) were female. The median community BP among these participants was SBP 125 mmHg/DBP 79 mmHg compared to the median clinic BP of SBP 146 mmHg/DBP 91 mmHg. Median community SBP was 21 mmHg lower and median DBP 8.5 mmHg lower than corresponding clinic measures. Of the 220 adults with isolated elevated BP in the community, the median age was 46 (IQR: 36–57) with 124 (56%) females. The median community BP was SBP 141 mmHg/DBP 92 mmHg, and the median clinic BP was SBP 126 mmHg/DBP 78 mmHg. Both median community SBP measurements and DBP measurements were 15 mmHg higher than clinic SBP and DBP measurements.

### Factors associated with concordant and discordant blood pressure measurements

Older age was associated with higher BP across settings (Table 3). Participants aged 40–65 were more likely to have concordant HTN than concordant normotension (PR 6.6 [4.9–9.1]), as well as participants aged 65 and older (PR 8.8 [6.1–12.5]) compared to ages 18–39 years. Participants aged 40–65 and participants 65 and older were more likely to have isolated clinic elevated BP than concordant normotension (40–65: PR 4.3 [2.3–8.1], 65 and older: PR 8.4 [4.0–18.2]). Participants aged 40–65 and participants 65 and older were also more likely to have isolated community elevated BP (40–65: PR 2.8 [2.0–3.9], 65 and older: PR 3.5 [2.0–5.9]).

Lower educational attainment was associated with concordant HTN and isolated clinic elevated BP. Participants with no education or only primary education, compared to secondary or higher education, were more likely to have concordant HTN (no education: PR 1.6 [1.2–2.0], primary: PR 1.4 [1.1–1.7]), and more likely to have isolated clinic elevated BP (no education: PR 3.0 [1.6–5.6], primary: PR 2.5 [1.4–4.3]). Overweight and obese BMIs were associated concordant HTN (overweight: PR 1.4 [1.1–1.7]), obese: PR 1.7 [1.4–2.1]), and with isolated community elevated BP (overweight: PR 1.4 [1.0–2.0] obese: PR 2.2 [1.5–3.1]).

## Discussion

This study compares community- and clinic-based BP measurements in a population-based cohort in urban Haiti. We found that our screening estimates of HTN prevalence were remarkably similar using community and clinic BP measurements, and BP measurements were highly correlated for both SBP and DBP. At the individual level, 85% of participants had concordant BP measurement classifications, with 59% classified as normotensive and 26% as hypertensive consistently across the two settings. These findings underscore the accuracy, reliability, and utility of using community-based BP measurements in a low-income setting to both estimate HTN prevalence and to identify patients with HTN.

The correlation of BP measurements from the community and the clinic was high, as was the concordance of BP status classification with either normotension or HTN diagnoses. This may reflect that BP measurements were performed consistently by similarly trained staff regardless of whether they were a CHW, nurse, or nursing aid. GHESKIO spent extensive time training staff in BP measurement with a mix of didactic lectures and hands-on learning, followed by repeated competency assessments and refresher trainings which all staff had to pass prior to study implementation. The high degree of concordance may also reflect less BP variation among urban Haitians than reported among other populations in high-income countries.

Interestingly, we did find that prevalence of HTN was slightly higher in community screening, as was the average community BP measurement. This finding may be partially explained by the order of measurements in the study, with community BP consistently measured before clinic BP in all participants. It is well established that BP measures tend to decrease over repeated readings and visits due to regression to the mean and familiarity with measurement procedures [25, 26]. Furthermore, CHWs provided education to participants after taking their community BP which could have led to behavioral changes influencing the subsequent clinic BP reading. The effect of behavioral modification is likely to be limited however given that most of the clinic measurements occurred within one week of the initial community measure. An alternative explanation for higher community BP in Haiti may be contextual differences between the community and clinic settings which may influence BP, such as stress, emotional state, conflict and perceived control [27–29]. For example, it is possible that the GHESKIO clinic was considered safe and calming in the setting of a highly unstable and stressful political and civil environment with extremely poor and disrupted access to health care. Further research is needed to identify if the slightly higher community BP measurements are due to the natural time course of repeated BP measurements or to contextual factors related to place of collection.

In cases where community BP measurements were elevated and clinic BP was normal, this may be a harbinger of potential masked HTN which is associated with similar if not worse CVD outcomes compared to persons with concordant HTN [30, 31]. Masked HTN is defined as BP measurement that is normal in the clinic but elevated in an out-of-office setting, as determined by ambulatory or home BP measurements [15, 21]. In a large multinational study comparing ambulatory BP with clinic measurements across participants from 11 high-income countries, individuals with masked HTN had an adjusted hazard ratio of 2.02 (95% CI 1.40–2.90) of cardiovascular events in comparison to normotensives [30]. While specific guidelines for treatment of masked HTN are evolving, current recommendations suggest that these individuals need clinical follow-up [21]. In low-income settings such as Haiti with limited access to clinic-based care and monitoring, elevated BP measurements in the community could be considered as an indicator for initiating antihypertensive medication without the requirement for confirmed clinic-based BP measurement, and in turn improve the treatment gap seen in many LMICs [32].

Community-based screening and treatment programs have been widely adopted for communicable diseases such as HIV for decades and are increasingly being utilized for non-communicable disease management in other LMICs [33–35]. Multiple studies have demonstrated that utilization of non-physician healthcare workers has been shown to improve HTN control by 50–70% and lower SBP by ~5 mmHg [36–38]. Unfortunately, there is limited data on the implementation of these programs for HTN management in Haiti or the Caribbean thus far, though our findings suggest initial efficacy of community-based screening. This is especially promising given that community BP is a significant predictor for ultimate CVD mortality. In a meta-analysis of eight prospective studies using community BP among 17,698 participants in Europe and Asia, increased community BP significantly predicted CVD mortality, more so than increased clinic BP [31]. The hazard ratio for all-cause mortality per 10 mmHg increase in SBP at community was 1.14 (95% CI 1.01–1.29) compared to 1.07 for the same increase in the clinic (95% CI 0.97–1.26).

Community BP screening may be especially useful for certain at-risk sub-populations who could be considered for initiation of treatment in the community even in the absence of a confirmatory clinic measure. For example, our data suggests that the majority of patients meeting the threshold of stage 2 HTN in the community remain hypertensive at the 140/90 cutoff in the clinic (86%), so community BP measurements may be used to initiate antihypertensive treatment for these individuals. This could suggest an actionable threshold of 160/100 to treat in the community in the presence of significant barriers to care such as transportation, clinic hours, and local violence. In the United States, HTN may be diagnosed based on a single BP measure of 180/120 in the absence of repeated measures, but given the limited resources in Haiti, it may be reasonable to treat at a lower threshold. Our findings suggest a high likelihood of confirmatory diagnosis in the clinic at this 160/100 threshold, indicating that this follow up may be unnecessary especially with the increased risk of the patient not returning to clinic for a confirmatory measure.

A strength of this study is that it is among the first to compare BP measurements in a community and clinic setting using a large population-based sample of adults in the Caribbean. Furthermore, study staff were extensively trained in BP measurement using international guidelines lending to the integrity and comparability of our results to international cohorts. Notably, the blood pressure devices used in the community and clinic were from the same company (OMRON) but differed slightly due to the necessity of employing a handheld device for community use. Although BP measurement procedures were identical and the machines were manufactured and validated by the same company, the different devices may be a source of discordance across the two settings as well. Ambulatory BP measurement was not available to establish diagnosis of masked or white-coat HTN [21], and the study did not capture nighttime BP which has been an established as one of the best predictors of CVD risk [15, 39]. Further research is needed to estimate the true prevalence of white-coat and masked HTN to determine how home BP measurements can be used to initiate treatment in these vulnerable patients.

In summary, we found high concordance of BP measurement classifications using community and clinic BP measurements in a population-based cohort in urban Haiti. These findings underscore the feasibility and accuracy of community BP measurement for community hypertension screening programs in LMICs. We encourage community-based BP screening programs as a practical public health strategy for improving hypertension screening, diagnosis, and treatment initiation. Expansion of community BP measurement in LMICs can improve access to hypertension screening and management and ultimately improve CVD-related health outcomes.

## Supplementary Material

Inclusivity in global research checklist S1 Checklist. Inclusivity in global research.

## Figures and Tables

**Table 1.** Characteristics of participants in the Haiti CVD cohort study (N = 2123).

	N (%)
Age, years	
Median [IQR; range]	41 [28–55; 18–90]
18–29	583 (27.5%)
30–39	410 (17.6%)
40–49	378 (17.8%)
50–59	358 (16.9%)
60+	394 (18.6%)
**Sex**	
Female	1320 (62.2%)
Male	803 (37.8%)
**Education**	
None	310 (14.6%)
Primary	482 (22.7%)
Secondary or Higher	1328 (62.6%)
Missing	3 (<1.0%)
**Income (daily)**	
≤1 USD	1511 (71.2%)
>1 USD	609 (28.7%)
Missing	3 (<1.0%)
**BMI (kg/m**^**2**^)	
Median (IQR)	24.4 (21.1–28.5)
Underweight / Normal <25.0	1150 (54.1%)
Overweight 25.0–29.9	580 (27.8%)
Obese ≥ 30.0	392 (18.5%)
**Smoking Status**	
Never	1928 (90.8%)
Current/Former	182 (8.6%)
Missing	13 (<1.0%)

**Table 2.** Blood pressure measurements of participants in the Haiti CVD cohort study (N = 2123).

	Community BP Measure	Clinic BP Measure
Blood pressure categories, n (%)		
Normal Pressure (SBP < 120 and DBP < 80)	800 (37.7%)	1024 (48.2%)
Pre-Hypertension (SBP 120–139 or DBP 80–89)	553 (26.0%)	455 (21.4%)
Hypertension (SBP ≥ 140 or DBP ≥ 90)	770 (36.3%)	644 (30.3%)
**Blood pressure measurements (mmHg)**		
SBP, Median (IQR)	122 (109–140)	118 (107–138)
DBP, Median (IQR)	80 (71–90)	73 (63–85)

The median number of days between home and clinic measurements was 4 ([IQR]: 2–11 days)

**Table 3.** Multivariable logistic regression models assessing factors associated with isolated elevated clinic blood pressure, isolated elevated community blood pressure, and concordant hypertension compared to participants with concordant normotension.

	Isolated Clinic Elevated BP	Isolated Community Elevated BP	Concordant Hypertension
	N = 1353	N = 1479	N = 1809
	(PR [95% CI])		
**Age Categories (years)**			
18–39	1 (ref)	1 (ref)	1 (ref)
40–65	**4.3 [2.3–8.1]** *	**2.8 [2.0–3.9]** *	**6.6 [4.9–9.1]** *
65+	**8.4 [4.0–18.2]** *	**3.5 [2.0–5.9]** *	**8.7 [6.1–12.5]** *
**Sex**			
Female	1 (ref)	1 (ref)	1 (ref)
Male	0.9 [0.6–1.5]	**0.7 [0.5–0.9]** *	0.8 [0.7–1.0]
**BMI category (kg/m**^**2**^)			
Normal or Underweight	1 (ref)	1 (ref)	1 (ref)
Overweight	1.4 [0.9–2.3]	**1.4 [1.0–12.0]** *	**1.4 [1.1−1.7]** *
Obese	1.4 [0.7–2.5]	**2.1 [1.5–3.1]** *	**1.7 [1.4−2.1]** *
**Education**			
Secondary or Higher	1 (ref)	1 (ref)	1 (ref)
Primary	**2.5 [1.4–4.3]** *	1.1 [0.8–1.6]	**1.4 [1.1–1.7]** *
None	**3.0 [1.6–5.6]** *	1.2 [0.8–2.0]	**1.6 [1.2–2.0]** *
**Income (Daily)**			
>1 USD	1 (ref)	1 (ref)	1 (ref)
≤1 USD	1.1 [0.7–1.6]	0.9 [0.6–1.2]	0.9 [0.7–1.1]
**Smoking Status**			
Never	1 (ref)	1 (ref)	1 (ref)
Current/Former	0.8 [0.3–1.7]	[0.8–1.9]	1.0 [0.7–1.3]

* = Significant p-values <0.05

Clinic elevated BP defined as clinic BP measurement ≥ 140/90 and community BP measurement < 140/90 Community elevated BP defined as community BP measurement ≥ 140/90 and clinic BP measurement < 140/90

## Data Availability

Data contain potentially identifying and sensitive patient information. Deidentified data used for this analysis are available upon request after signing a data access and use agreement, provision of approval by the GHESKIO ethics board, and demonstration that the external investigative team is qualified and has documented evidence of human research protection training. Requests may be addressed to Jean William Pape, jwpape@gheskio.org, or irb@med.cornell.edu.

## References

[1] Hypertension. Accessed September 22, 2021. https://www.who.int/news-room/fact-sheets/detail/hypertension

[2] Global Action Plan for the Prevention and Control of NCDs 2013–2020. Accessed September 22, 2021. https://www.who.int/publications-detail-redirect/9789241506236

[3] Chow CK, Teo KK, Rangarajan S, Islam S, Gupta R, Avezum A, Prevalence, awareness, treatment, and control of hypertension in rural and urban communities in high-, middle-, and low-income countries. JAMA. 2013; 310(9):959–968. 10.1001/jama.2013.184182 24002282

[4] Verberk WJ, Kroon AA, Kessels AGH, de Leeuw PW. Home Blood Pressure Measurement: A Systematic Review. Journal of the American College of Cardiology. 2005; 46(5):743–751. 10.1016/j.jacc.2005.05.058 16139119

[5] Gaziano TA, Abrahams-Gessel S, Denman CA, Montano CM, Khanam M, Puoane T, An assessment of community health workers’ ability to screen for cardiovascular disease risk with a simple, non-invasive risk assessment instrument in Bangladesh, Guatemala, Mexico, and South Africa: an observational study. Lancet Glob Health. 2015; 3(9):e556–563. 10.1016/S2214-109X(15)00143-6 26187361 PMC4795807

[6] Ndip Agbor V, Temgoua MN, Noubiap JJN. Scaling up the use of home blood pressure monitoring in the management of hypertension in low-income countries: A step towards curbing the burden of hypertension. J Clin Hypertens (Greenwich). 2017; 19(8):786–789. 10.1111/jch.12999 28371238 PMC8031063

[7] IHE/Haiti IH de l’Enfance-, ICF. Haiti Enquête Mortalité, Morbidité et Utilisation des Services 2016–2017—EMMUS-VI. Published online July 1, 2018. Accessed September 10, 2021. https://www.dhsprogram.com/publications/publication-fr326-dhs-final-reports.cfm

[8] Tymejczyk O, McNairy ML, Petion JS, Rivera VR, Dorélien A, Peck M, Hypertension prevalence and risk factors among residents of four slum communities: population-representative findings from Port-au-Prince, Haiti. Journal of Hypertension. 2019; 37(4):685–695. 10.1097/HJH.0000000000001966 30817448 PMC7680636

[9] McNairy ML, Tymejczyk O, Rivera V, Seo G, Dorélien A, Peck M High Burden of Non-communicable Diseases among a Young Slum Population in Haiti. J Urban Health. 2019; 96(6):797–812. 10.1007/s11524-019-00368-y 31218502 PMC6904710

[10] Liu K, Ballew C, Jacobs DR, Sidney S, Savage PJ, Dyer A, Ethnic differences in blood pressure, pulse rate, and related characteristics in young adults. The CARDIA study. Hypertension. 1989; 14 (2):218–226. 10.1161/01.hyp.14.2.218 2759681

[11] The Politics of Violence in Latin America. Vol 15. 1st ed. University of Calgary Press; 2019. 10.2307/j.ctvkwnpz9

[12] Kwan GF, Yan LD, Isaac BD, Bhangdia K, Jean-Baptiste W, Belony D, High Poverty and Hardship Financing Among Patients with Noncommunicable Diseases in Rural Haiti. Glob Heart. 2020; 15(1):7. 10.5334/gh.388 32489780 PMC7218772

[13] Pape JW, Rouzier V, Ford H, Joseph P, Johnson WD, Fitzgerald DW. The GHESKIO Field Hospital and Clinics after the Earthquake in Haiti—Dispatch 3 from Port-au-Prince. New England Journal of Medicine. 2010; 362(10):e34. 10.1056/NEJMpv1001787 20164476

[14] Piette JD, Datwani H, Gaudioso S, Foster SM, Westphal J, Perry W, Hypertension management using mobile technology and home blood pressure monitoring: results of a randomized trial in two low/middle-income countries. Telemed J E Health. 2012; 18(8):613–620. 10.1089/tmj.2011.0271 23061642 PMC4361160

[15] Pickering TG, Eguchi K, Kario K. Masked hypertension: a review. Hypertens Res. 2007; 30(6):479–488. 10.1291/hypres.30.479 17664850

[16] Anderson C, Dadabhai S, Damasceno A, Dzudie A, Islam SMS, Kamath D, Home Blood Pressure Management Intervention in Low- to Middle-Income Countries: Protocol for a Mixed Methods Study. JMIR Res Protoc. 2017; 6(10):e188. 10.2196/resprot.7148 29038099 PMC5662792

[17] Fleming S, Atherton H, McCartney D, Hodgkinson J, Greenfield S, Hobbs FDR, Self-Screening and Non-Physician Screening for Hypertension in Communities: A Systematic Review. Am J Hypertens. 2015; 28(11):1316–1324. 10.1093/ajh/hpv029 25801901 PMC4506785

[18] Gill P, Haque MS, Martin U, Mant J, Mohammed MA, Heer G, Measurement of blood pressure for the diagnosis and management of hypertension in different ethnic groups: one size fits all. BMC Cardiovascular Disorders. 2017; 17(1):55. 10.1186/s12872-017-0491-8 28178928 PMC5299651

[19] Lookens J, Tymejczyk O, Rouzier V, Smith C, Preval F, Joseph I, The Haiti cardiovascular disease cohort: study protocol for a population-based longitudinal cohort. BMC Public Health. 2020; 20(1):1633. 10.1186/s12889-020-09734-x 33131500 PMC7603639

[20] World Health Organization. Noncommunicable Diseases and Mental Health Cluster. WHO STEPS Surveillance Manual: The WHO STEPwise Approach to Chronic Disease Risk Factor Surveillance. World Health Organization; 2005. Accessed September 10, 2021. https://apps.who.int/iris/handle/10665/43376

[21] Whelton PK, Carey RM, Aronow WS, Casey DE, Collins KJ, Himmelfarb CD, 2017 ACC/AHA/AAPA/ABC/ACPM/AGS/APhA/ASH/ASPC/NMA/PCNA Guideline for the Prevention, Detection, Evaluation, and Management of High Blood Pressure in Adults. Journal of the American College of Cardiology. 2018; 71(19):e127–e248. 10.1016/j.jacc.2017.11.006 29146535

[22] Juraschek SP, Ishak AM, Mukamal KJ, Wood JM, Anderson TS, Cohen ML, Impact of 30- Versus 60-Second Time Intervals Between Automated Office Blood Pressure Measurements on Measured Blood Pressure. Hypertension. 2021; 78(5):1502–1510. 10.1161/HYPERTENSIONAHA.121.17876 34488436 PMC8715230

[23] Takahashi H, Yoshika M, Yokoi T. Validation of two automatic devices for the self-measurement of blood pressure according to the ANSI/AAMI/ISO81060-2:2009 guidelines: the Omron BP765 (HEM-7311-ZSA) and the Omron BP760N (HEM-7320-Z). Vasc Health Risk Manag. 2015; 11:49–53. 10.2147/VHRM.S72438 25657587 PMC4295899

[24] Ahmad OB, Boschi Pinto C, Lopez A, Murray C, Lozano R, Inoue M. Age Standardization of Rates: A New WHO Standard. GPE Discussion Paper Series, EIP/GPE/EBD, World Health Organization. 2001; No.31.

[25] Moore MN, Atkins ER, Salam A, Callisaya ML, Hare JL, Marwish TH, Regression to the mean of repeated ambulatory blood pressure monitoring in five studies. Journal of Hypertension. 2019; 37 (1):24–29. 10.1097/HJH.0000000000001977 30499921

[26] Morton V, Torgerson DJ. Effect of regression to the mean on decision making in health care. BMJ. 2003; 326(7398):1083–1084. 10.1136/bmj.326.7398.1083 12750214 PMC1125994

[27] Tomitani N, Kanegae H, Suzuki Y, Kuwabara M, Kario K. Stress-Induced Blood Pressure Elevation Self-Measured by a Wearable Watch-Type Device. American Journal of Hypertension. 2021; 34 (4):377–382. 10.1093/ajh/hpaa139 32852527 PMC8057129

[28] Kamarck TW, Janicki DL, Shiffman S, Polk DE, Muldoon MF, Liebenauer LL, Psychosocial demands and ambulatory blood pressure: a field assessment approach. Physiol Behav. 2002; 77(4–5):699–704. 10.1016/s0031-9384(02)00921-6 12527022

[29] Kamarck TW, Schwartz JE, Shiffman S, Muldoon MF, Sutton-Tyrrell K, Janicki DL. Psychosocial Stress and Cardiovascular Risk: What is the Role of Daily Experience? Journal of Personality. 2005; 73 (6):1749–1774. 10.1111/j.0022-3506.2005.00365.x 16274452

[30] Franklin SS, Thijs L, Hansen TW, Li Y, Boggie J, Kikuya M, Significance of white-coat hypertension in older persons with isolated systolic hypertension: a meta-analysis using the International Database on Ambulatory Blood Pressure Monitoring in Relation to Cardiovascular Outcomes population. Hypertension. 2012; 59(3):564–571. 10.1161/HYPERTENSIONAHA.111.180653 22252396 PMC3607330

[31] Ward AM, Takahashi O, Stevens R, Heneghan C. Home measurement of blood pressure and cardiovascular disease: systematic review and meta-analysis of prospective studies. Journal of Hypertension. 2012; 30(3):449–456. 10.1097/HJH.0b013e32834e4aed 22241136

[32] Geldsetzer P, Manne-Goehler J, Marcus ME, Ebert C, Zhumadilov Z, Wesseh CS, The state of hypertension care in 44 low-income and middle-income countries: a cross-sectional study of nationally representative individual-level data from 1 · 1 million adults. The Lancet. 2019; 394(10199):652–662. 10.1016/S0140-6736(19)30955-9 31327566

[33] Kredo T, McCaul M, Volmink J. Task-shifting from doctors to non-doctors for initiation and maintenance of antiretroviral therapy. S Afr Med J. 2015; 105(8):626–627. 10.7196/samj.10091 26449702

[34] Eberly LA, Rusangwa C, Ng’ang’a L, Neal CC, Mukundiyukuri JP, Mpanusingo E, Cost of integrated chronic care for severe non-communicable diseases at district hospitals in rural Rwanda. BMJ Glob Health. 2019; 4(3):e001449. 10.1136/bmjgh-2019-001449 PMC6597643 31321086

[35] Mercer T, Nulu S, Vedanthan R. Innovative Implementation Strategies for Hypertension Control in Low- and Middle-Income Countries: a Narrative Review. Curr Hypertens Rep. 2020; 22(5):39. 10.1007/s11906-020-01045-1 32405820

[36] Jafar TH, Gandhi M, de Silva HA, Jehan I, Naheed A, Finkelstein EA, A Community-Based Intervention for Managing Hypertension in Rural South Asia. N Engl J Med. 2020; 382(8):717–726. 10.1056/NEJMoa1911965 32074419

[37] He J, Irazola V, Mills KT, Poggio R, Beratarrechea A, Dolan J, Effect of a Community Health Worker-Led Multicomponent Intervention on Blood Pressure Control in Low-Income Patients in Argentina: A Randomized Clinical Trial. JAMA. 2017; 318(11):1016–1025. 10.1001/jama.2017.11358 28975305 PMC5761321

[38] Anand TN, Joseph LM, Geetha AV, Prabhakaran D, Jeemon P. Task sharing with non-physician health-care workers for management of blood pressure in low-income and middle-income countries: a systematic review and meta-analysis. The Lancet Global Health. 2019; 7(6):e761–e771. 10.1016/S2214-109X(19)30077-4 31097278 PMC6527522

[39] Hodgkinson J, Mant J, Martin U, Guo B, Hobbs FDR, Deeks JJ, Relative effectiveness of clinic and home blood pressure monitoring compared with ambulatory blood pressure monitoring in diagnosis of hypertension: systematic review. BMJ. 2011; 342:d3621. 10.1136/bmj.d3621 21705406 PMC3122300

